# Prevalence and associated factors of mortality after percutaneous coronary intervention for adult patients with ST elevation myocardial infarction: A systematic review and meta-analysis protocol

**DOI:** 10.1097/MD.0000000000016226

**Published:** 2019-06-28

**Authors:** Fanghong Yan, Huan Liu, Wenhui Jiang

**Affiliations:** a Xi’an Jiaotong University Health Science Center, Shaanxi Province; b Lanzhou University, School of Nursing, Gansu Province, China.

**Keywords:** factors, mortality, percutaneous coronary intervention, prevalence, ST elevation myocardial infarction

## Abstract

**Background:**

The percutaneous coronary intervention (PCI) has been one of the fastest growing therapeutic interventions for patients with ST elevation myocardial infarction (STEMI). However, the mortality of patients with STEMI after PCI is uncertain currently. There is a paucity of systematic review on the associated factors of mortality among patients with STEMI after PCI. Therefore, this meta-analysis was designed to synthesize available evidence on the prevalence and associated factors of mortality after PCI for adult patients with STEMI.

**Methods:**

Both case–control and cohort studies reporting on mortality after PCI for patients with STEMI, published in Chinese and English will be eligible for inclusion. Studies from 12 databases covering the period from 2008 to present will be considered for systematic searches. Two reviewers will independently screen and select studies, extract data, and assess methodologic quality. When available, meta-analysis will be performed. Pooled proportions of mortality, and proportions in the exposed and unexposed groups, and population attributable fraction of each factor will be calculated by a suitable transformation of proportions. If necessary, meta-regression models, subgroup analysis, sensitivity analysis, funnel plot, and Egger test will be performed. Narrative synthesis will be done where meta-analysis cannot be performed. Reporting of this protocol will comply with the preferred reporting items for systematic review and meta-analyses (PRISMA-P) guidelines.

**Results:**

This systematic review will be developed according to the meta-analysis of observational studies in epidemiology (MOOSE) guidelines.

**Conclusion:**

This study will provide a comprehensive review on the available evidence regarding the prevalence and associated factors of mortality for patients with STEMI following PCI. This review will be constrained by the divergence of definition and assessment of specific factors between studies. However, the development of a qualitative description of definition and assessment tools will also provide an overview of the current practice. Formal ethical approval is not required since the secondary data will be collected for systematic review. The findings will be disseminated in a relevant peer-reviewed journal and academic presentations.

**Systematic review registration:**

PROSPERO CRD42017070969

## 1 Introduction

The prevalence of the cardiovascular disease has rapidly and continuously risen in the past decades,^[1]^ leading to tremendous disease burden worldwide.^[2,3]^ Among them, acute coronary syndrome (ACS), consisting of ST-segment elevation myocardial infarction (STEMI), non-STEMI and unstable angina, accounts for almost half of the overall mortality related to cardiovascular disease.^[2]^ Of all ACS, STEMI, as a high-risk medical emergency, remains the highest cause of morbidity.^[4]^

Over the past 20 years period, one of the fastest growing therapeutic interventions for patient with STEMI is percutaneous coronary intervention (PCI), which is the most common technique to improve myocardial perfusion.^[5,6]^ PCI appears to be now the dominant treatment of STEMI in the majority of countries, especially in China. Almost half a million PCIs were performed each year with a steady-state growth rate from 2010 to 2015.^[7]^ Even though PCI is very efficacious in improving symptoms for patients with angina in STEMI, there are risks of adverse cardiac events and even death for patients with STEMI after PCI. However, whether the progress has translated into improved survival of patients with STEMI is not very clear. Most nationwide studies report a concomitant decrease in mortality for unselected patients with STEMI, which is not accurate enough to assess the improvement.^[8–10]^ There is considerable variability of the mortality (1.5–48.1%) among studies with a 1-year follow-up after PCI.^[11,12]^ Therefore, it is necessary to systematically synthesize the mortality rate for patients with STEMI following PCI.

Furthermore, identification of the highest risk patients with STEMI is very important for providing individually focused management and improve prognosis.^[13]^ However, no systematic review has been published on the related factors of mortality among patients with STEMI after PCI. Therefore, the aim of this systematic review and meta-analysis is designed to critically synthesize published data to report the mortality rate and screen associated factors, to provide basic knowledge for designing cost-effective prevention program to control the factors, and further improve prognosis and decrease mortality in adult patients with STEMI following PCI.^[14,15]^

## 2 Research objectives

To conduct a systematic review and meta-analysis to identify the prevalence of mortality after PCI for adult patients with STEMI with different follow-up period, as well as associated factors. The absolute risk, odds ratio (OR), and population attributable fraction (PAF) of each specific factor improving outcome will be estimated between exposed and unexposed groups.

## 3 Methods

This systematic review and meta-analysis will be developed according to the meta-analysis of observational studies in epidemiology (MOOSE) guidelines.^[16]^ Reporting of this protocol will comply with the preferred reporting items for systematic review and meta-analyses protocols (PRISMA-P) 2015 guidelines.^[17]^ This review has been registered on PROSPERO international prospective register of systematic review, registration number (CRD 42017070969).^[18]^ In case of any revision of this protocol after publication, the content and rationale will be published in the PROSPERO registry.

### 3.1 Inclusion criteria

#### 3.1.1 Study population

The study population will be confirmed STEMI adult patients (>19 years old) after PCI diagnosed by a medical healthcare provider. STEMI was defined according to the criteria recommended by the American College of Cardiology and European Society of Cardiology guidelines. STEMI was diagnosed when ST-segment elevation ≥1 mm was seen in at least 2 contiguous leads in any location on the index or qualifying Electrocardiograph, or when presumed new left bundle-branch block or documented new Q waves were observed.^[19]^

#### 3.1.2 Study settings

Population-based or hospital-based studies.

#### 3.1.3 Type of studies

Both case–control and cohort studies when they meet the inclusion criteria.

#### 3.1.4 Types of outcomes

Death included cardiac and noncardiac death.

#### 3.1.5 Exposure of interest

In this context, factors associated with mortality are attributes characteristics or exposures that will increase the likelihood of mortality for patients with STEMI after PCI, which can be negatively (hazardous factor) and positively (protective factor). Prognostic factors will be categorized into 5 groups and included, but are not limited to, the following^[20]^:

- Environmental factors (e.g., medications not taken for beta-blockers, smoking status, and alcohol intake)
- Social factors (e.g., employment, educational level, and socioeconomic status),
- Biologic factors (e.g., comorbidities)
- Genetic factors (e.g., age and race)
- Disease factors (e.g., infarction area, symptom-onset-to-balloon time, target vessel).

#### 3.1.6 Date of publication

Studies from databases covering the period from 2008 to present will be considered for this review.

#### 3.1.7 Language of publications

English and Chinese language will be included as this review focuses on study reporting.

#### 3.1.8 Type of publications

To avoid publication bias, all relevant studies, including journal papers, dissertation and conference abstracts will be included. For conference abstracts, it is necessary to ensure that the data are same with corresponding full reports. When there is a need for more details, authors will be contacted.

### 3.2 Exclusion criteria

#### 3.2.1 Study population

- Patients who underwent another surgery during the same hospitalization.

#### 3.2.2 Type of study

- Letters, reviews, commentaries, editorials, meta-analysis researches, cross-sectional studies, qualitative researches.
- Studies lacking a specific number of outcomes for quantitative analysis and explicit method description.
- Studies from which key information cannot be obtained even after a request from authors.
- Duplicates: studies with the same data, of which the one reporting the largest sample size will be considered.
- Ongoing studies with the interim result.
- Studies dealing with a small sample size (fewer than 20 participants).
- Studies with the aim to assess the impact of indicators associated with genetic polymorphism.
- Studies associated with a specific factor for which there are ≤2 studies.

### 3.3 Search strategy and literature sources

A 3-step search strategy will be utilized in this review. An initial limited search of the JBI Database of Systematic Reviews and Implementation Reports, the Cochrane Library of Systematic Reviews, MEDLINE, PROSPERO, and Embase will be undertaken, followed by an analysis of the text words contained in the title and abstract, and the index terms used to describe articles. A subsequent search using all identified keywords and index terms will then be performed across all included databases. Last, the reference list of all identified reports and articles will be searched for further studies of interest. When we cannot access a full-text publication for free, authors will be contacted.

The databases to be searched include the following: Embase via Embase.com, Medline via OVID, Scopus, China National Knowledge Infrastructure, China Science and Technology Journal Database, Chinese Biomedical Database, WanFang Data, ProQuest Dissertations and Theses, ClinicalTrials.gov, Chinese Clinical Trial Register, China Master's Theses Full-text Databases and China Doctoral Dissertation Full-text Database.

### 3.4 Search language

The “PICO” (population, intervention, comparator, and outcome) framework will be used to construct an effective combination of search terms. Since this review concentrates on the prevalence and factors of mortality, and factors are not set in advance, the search strategy will only comprise population (STEMI and PCI), study design (case–control and cohort studies), and outcome (mortality). Boolean operators “OR” will be used to combine Subject Headings and synonyms of free-text words relating to specific terms, then “AND” will be used to combine the results of different terms. As there are multiple forms of terms in articles on this subject, we will construct a search strategy as extensive as possible. Search strategies of “STEMI,” “PCI,” and “mortality” will be drawn on the experience of published systematic reviews. For the design of researches, published search filters for various databases from The Centre for Review and Dissemination will be applied in this review.^[21]^

Furthermore, these limits of age filter (>19 years old), language filter (English or Chinese) and publication period (2008-present) will be applied to available databases. Table 1 shows the search strategy in MEDLINE via OVID, which will be revised to fit Embase, ProQuest Dissertations and Theses, Scopus and ClinicalTrials.gov. For Chinese databases, 3 terms (STEMI, PCI, and mortality) will be used to structure the search since subject headings search and free-text search are both unable to distinguish the design of studies exactly.

**Table 1:**
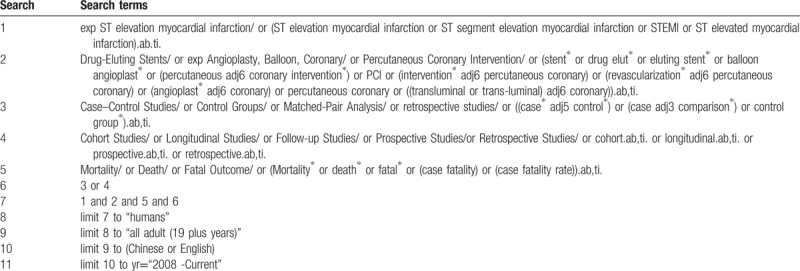
Search strategy in MEDLINE via OVID.

### 3.5 Data collection

#### 3.5.1 Documenting the search process

The search process will be documented and reported as supplemental materials to ensure that we can rerun the entire process later. The date searched, name of the database, the website of a search engine, search terms, and the number of records will be recorded in a form.

#### 3.5.2 Study selection

Records emerging from the above search strategy will be transferred to Endnote X7 for papers in English and NoteExpress 3.2 for papers in Chinese. First, apparent duplicate studies will be removed with preference settings of the same author, publication year and title. Then, 2 review authors (FHY and HL) will independently screen titles and abstracts based on the same inclusion and exclusion criteria to exclude obviously nonrelevant records. During this stage, the overinclusion might be allowed. Records approved by either author will be included in the last stage of full text reviews. The same authors will independently inspect and further assess full text articles. Any discrepancies will be resolved by a third author (Jiang). For all identified reports and articles, reference lists will be screened for further studies of interest. At last, the selection process will be summarized with the use of a flow chart in accordance with the PRISMA framework.

#### 3.5.3 Piloting the study search and selection process

Firstly, search strategies will be applied to Medline, with the search period of 3 months. Then we will pilot the selection process to above records to check whether the inclusion criteria can be reliably interpreted to get identified papers appropriately. During this process, the expression of inclusion criteria will also be refined to improve the consistency between the 2 reviewers (FHY and HL).

#### 3.5.4 Data extraction

A standardized template of a data collection form will be preliminarily designed and pilot-tested on 1 study of each type by the 2 authors (FHY and HL) to make sure all necessary information is included. The information to be captured will be categorized as follows: study characteristics (author, location, publication year), patient characteristics (STEMI definition, sample size, age, gender, the history of revascularization procedures), methods (study design, sampling approach, study period, information for the assessment of risk bias, etc), factors identified (associated factors, unadjusted data, definition, and measurement method), mortality (definition, data, and length of follow-up), the funding and notes. In the case of multiple papers with the same study population, data will be extracted from the most informative paper; however, if the papers are the same, the earliest year of publication will be adopted. If necessary, authors of original studies will be consulted for unclear or missing information. When the 2 authors independently extract data using the above form, any disagreement will be settled by discussion or 3rd author (WHJ).

#### 3.5.5 Assessment of methodologic quality

With the principle of a high threshold for exclusion, pairs of reviewers, working independently, and in duplicate, will select papers for full-text retrieval to 1st be assessed by 2 independent authors (FHY and HL) for methodologic validity prior to inclusion in the review. The Newcastle–Ottawa scale will be used for cohort studies and case–control studies.^[22]^ Any disagreements that arise between 2 reviewers will be settled through mutual discussion or a 3rd author (Jiang).

#### 3.5.6 Data analysis and synthesis

Quantitative data will, where possible, be pooled in statistical meta-analysis using R 3.4.3 software, and values of *P* < .05 are considered significant for all analyses. We calculate the pooled proportions to estimate the overall and specific prevalence of mortality with different follow-up period (≤30 days, 1–6 months, 0.5–1 year, 1–3 years, 3–5 years, ≥5 years) with a suitable transformation of proportions, which depends on the value of the Shapiro–Wilk normality test of untransformed proportions, logit transformation, log transformation, Freeman–Tukey double arcsine transformation, and arcsine transformation.

For each risk factor, the pooled proportion (*P*_pooled_) in the exposed and unexposed groups will also be calculated using the above procedures. Then we use a random effect model to calculate a pooled OR (OR_pooled_) and 95% confidence interval for each risk factor when a statistical test of heterogeneity is evident across studies. And lastly, the PAF for each risk factor will be calculated by the following formula under use of the *P*_pooled_ and OR_pooled_^[23]^:

PAF = [*P*_pooled_ (OR_pooled_ − 1)]/[*P*_pooled_ (OR_pooled_ − 1) + 1]

When 10 or more papers are included in a meta-analysis, the combined use of meta-regression and subgroup analysis will be performed to detect possible sources of substantial heterogeneity. Meta-regression models will be firstly undertaken while adjusting for relevant covariates (refer to age, gender, geographic setting, the funding, and sample size) to screen stratified factors, and then the subgroup analysis will be used to compare changes in heterogeneity before and after. When quality, study design and sample size of the included studies are inconsistent, sensitivity analysis will be performed to exclude the corresponding studies to estimate the effect. To assess publication bias, the funnel plot and Egger test will also be constructed.

The measures of heterogeneity of studies will utilize forest plot, Chi-squared, and *I*^2^ tests, where *I*^2^ values of 25%, 50%, and 75% represent low, medium, and high heterogeneity, respectively.^[24]^ Where statistical pooling is not sufficiently homogenous for combination, the findings will be presented in narrative form with tables and figures to aid in data presentation. At last, a summary table will be made using GRADEPro in terms of the grading of recommendations assessment, development, and evaluation (GRADE) guideline, which aims to assess the quality of evidence provided by systematic reviews.

#### 3.5.7 Patient and public involvement

Since this review is a synthesis of secondary public information, there is no need for patient and public involvement.

## 4 Discussion

This review will construct the comprehensive search strategy for 12 databases, including dissertation and conference abstracts to not miss any relevant studies, following the examination of the completeness and accuracy of reporting from current practice by a pilot-tested template. It will also synthesis the evidence on the prevalence of mortality after PCI for patients with STEMI, which are previously prescribed. The findings of this review also provide insight into the contributions of associated factors, considering the pooled proportions and OR between exposed and unexposed groups, and also the PAF as a metric applicable to public health initiatives at the population level.^[23]^ Furthermore, the application of complementary analysis, such as meta regression, subgroup analysis, sensitivity analysis, funnel plot, and Egger test can be effective to improve the quality and credibility of evidences provided by this review.

As we have set a language filter, we may miss some relevant papers published in other language. However, with the shift toward the publications of studies in English, the extent and effects of language bias may have recently been reduced.^[25,26]^ Moreover, this review will be limited in the divergence of definition and assessment of specific factors between studies. However, the development of a qualitative description of definition and assessment tools will also provide an overview of the current practice.

## Author contributions

JWH, YFH, and LH conceived and designed the protocol. YFH drafted the manuscript. JWH is the guarantor of the review. YFH, LH critically revised the manuscript for methodologic and intellectual content. All authors have both read and approved the final version of this manuscript.

**Conceptualization:** Fanghong Yan, Wenhui Jiang.

**Data curation:** Fanghong Yan, Huan Liu.

**Formal analysis:** Fanghong Yan, Huan Liu.

**Funding acquisition:** Wenhui Jiang.

**Investigation:** Fanghong Yan, Huan Liu.

**Methodology:** Fanghong Yan, Huan Liu.

**Project administration:** Fanghong Yan.

**Resources:** Wenhui Jiang.

**Software:** Fanghong Yan.

**Supervision:** Wenhui Jiang.

**Validation:** Wenhui Jiang.

**Visualization:** Fanghong Yan.

**Writing – original draft:** Fanghong Yan.

**Writing – review & editing:** Huan Liu, Wenhui Jiang.

Fanghong Yan orcid: 0000-0001-5763-8820.
